# Ectopic Expression of Retrotransposon-Derived PEG11/RTL1 Contributes to the Callipyge Muscular Hypertrophy

**DOI:** 10.1371/journal.pone.0140594

**Published:** 2015-10-16

**Authors:** Xuewen Xu, Fabien Ectors, Erica E. Davis, Dimitri Pirottin, Huijun Cheng, Frédéric Farnir, Tracy Hadfield, Noelle Cockett, Carole Charlier, Michel Georges, Haruko Takeda

**Affiliations:** 1 Unit of Animal Genomics, GIGA Research Center and Faculty of Veterinary Medicine, University of Liège, 1 Avenue de l’Hôpital, Liège, Belgium; 2 Transgenic platform, FARAH and GIGA Research Center, University of Liège, 1 Avenue de l’Hôpital, Liège, Belgium; 3 Unit of Biostatistics, FARAH and Faculty of Veterinary Medicine, University of Liège, Boulevard de Colonster, Liège, Belgium; 4 Department of Animal, Dairy and Veterinary sciences, Utah State University, Logan, Utah, United States of America; University of Minnesota Medical School, UNITED STATES

## Abstract

The callipyge phenotype is an ovine muscular hypertrophy characterized by polar overdominance: only heterozygous *+* ^*Mat*^ */CLPG* ^*Pat*^ animals receiving the *CLPG* mutation from their father express the phenotype. *+* ^*Mat*^ */CLPG* ^*Pat*^ animals are characterized by postnatal, ectopic expression of Delta-like 1 homologue (DLK1) and Paternally expressed gene 11/Retrotransposon-like 1 (PEG11/RTL1) proteins in skeletal muscle. We showed previously in transgenic mice that ectopic expression of DLK1 alone induces a muscular hypertrophy, hence demonstrating a role for DLK1 in determining the callipyge hypertrophy. We herein describe newly generated transgenic mice that ectopically express PEG11 in skeletal muscle, and show that they also exhibit a muscular hypertrophy phenotype. Our data suggest that both DLK1 and PEG11 act together in causing the muscular hypertrophy of callipyge sheep.

## Introduction

### The callipyge phenotype

The callipyge phenotype is a muscular hypertrophy described in sheep. The augmented muscle mass (less than or equal to 50% increase) characterizing callipyge animals results from the larger proportion and diameter of glycolytic, fast twitch type II muscle fibers. The callipyge phenotype manifests itself at approximately one month of age and is characterized by a rostro-caudal gradient (i.e. more pronounced in the posterior than in the anterior part of the body). Callipyge meat is tougher, and this has precluded its large-scale commercial use (reviewed in [1] and references therein).

### *Cis*-effects of the *CLPG* mutation

The callipyge phenotype is determined entirely by the *CLPG* mutation, an *A* to *G* transition in a highly conserved dodecamer motif located in the 90-Kb intergenic region separating the *Delta-like 1 homologue* (*DLK1*) and *Gene trap locus 2* (*GTL2*) genes in the eponymous imprinted domain [2, 3]. The *CLPG* mutation is thought to inactivate a muscle-specific silencer thereby causing prolonged, ectopic expression of *DLK1*, *GTL2*, *Paternally expressed gene 11/Retrotransposon-like 1* (*PEG11/RTL1*), *Antisense PEG11* (*anti-PEG11)*, *RNA imprinted and accumulated in nucleus* (*RIAN*), *miRNA containing gene* (*MIRG*) and associated small RNAs in postnatal skeletal muscle. The *CLPG* mutation does not affect imprinting control of the *DLK1-GTL2* domain: the protein-encoding *DLK1* and *PEG11* remain preferentially expressed from the paternal allele, while the non-coding long RNAs, *GTL2*, *anti-PEG11*, *RIAN*, and *MIRG*, with more than 47 C/D snoRNAs and more than 110 embedded microRNAs, remain preferentially expressed from the maternal allele (f.i. [4, 5]). The expression of the more distantly located *Brain-enriched guanylate kinase-associated* (*BEGAIN*) and *Deiodinase iodothyronine type III* (*DIO3*) imprinted genes in the same domain is not affected by the callipyge mutation [6]. As a result of this *cis*-effect of the callipyge mutation, the expression pattern of the affected core cluster of genes in the *DLK1-GTL2* domain is distinct in the four genotypes: (i) all target genes are silenced after birth in skeletal muscle of +/+ animals, (ii) all target genes are expressed ectopically in skeletal muscle of *CLPG/CLPG* animals, (iii) all maternally expressed (long and small) non-coding RNAs are expressed ectopically in skeletal muscle of *CLPG* ^*Mat*^ */+* ^*Pat*^ animals, and (iv) the paternally expressed protein encoding *DLK1* and *PEG11* genes are ectopically expressed in skeletal muscle of *+* ^*Mat*^ */CLPG* ^*Pat*^ animals (reviewed in [1]).

### *Trans*-effects underlying polar overdominance

The most remarkable feature of the callipyge phenomenon is the fact that only *+* ^*Mat*^ */CLPG* ^*Pat*^ animals display the muscular hypertrophy. This non-Mendelian mode of inheritance is referred to as polar overdominance [7]. It differs from regular parental imprinting as homozygous *CLPG/CLPG* animals do not express the phenotype. This is thought to result from the post-transcriptional *trans*-inactivation of the paternally expressed protein-coding *DLK1* and *PEG11* genes by one or more of the long and/or small non-coding maternally expressed RNAs from the domain [8]. As a consequence, DLK1 and PEG11 are expressed at the protein level only in postnatal skeletal muscle of *+* ^*Mat*^ */CLPG* ^*Pat*^ animals, the only individuals expressing the callipyge phenotype [9–11]. The *trans*-inactivation of *PEG11* in *CLPG/CLPG* animals has been shown to result from the RISC-mediated cleavage of *PEG11* mRNA by at least three perfectly complementary miRNAs (oar-*miR-136*, *-127* and *-432*) processed from the maternally expressed *anti-PEG11* non-coding RNA [12]. The molecular mechanisms underlying the *trans*-inactivation of *DLK1* remain elusive [13].

### Role of DLK1 and/or PEG11 protein in causing the callipyge phenotype

The distinctive features of *+* ^*Mat*^ */CLPG* ^*Pat*^ animals (i.e. (i) the only genotype expressing the callipyge phenotype, and (ii) the only genotype expressing DLK1 and PEG11 protein in skeletal muscle after birth), strongly suggest that ectopic expression of DLK1 and/or PEG11 protein causes the callipyge muscular hypertrophy. DLK1 encodes a member of the Notch-Delta family of membrane receptors/ligands, which is known to be involved in adipogenesis, haematopoiesis, neurogenesis and adaptation to independent life (f.i. [1]). DLK1 has been implicated directly in causing the callipyge muscular hypertrophy in a murine model; transgenic mice expressing ovine *DLK1* (C2 isoform) under the dependence of the muscle specific murine *Myosin light chain* 3F promoter and 2E enhancer (*Mlc* 3F/2E) exhibit a muscular hypertrophy [9].

PEG11 is an “exapted” protein derived from a eutherian-specific *sushi*-*ichi* retrotransposon, which is essential for the maintenance of the feto-maternal placental interface [14]. Notably, enhanced expression of PEG11 was shown recently to drive hepatocarcinogenesis [15]. Whether PEG11 participates in producing the callipyge phenotype has remained undetermined so far.

Here, we describe the generation of transgenic mice expressing ovine *PEG11* under the dependence of the same *Mlc* 3F/2E promoter and enhancer combination to study the contribution of PEG11 in producing the callipyge phenotype.

## Results

### Generating transgenic mice expressing ovine *PEG11* in skeletal muscle

First, we cloned the ovine *PEG11* open reading frame (*oPEG11* ORF), encoding 1,333 amino acids, into a modified pMlc3F-nlacz-2E vector, placing it under the dependence of the *Mlc* 3F/2E [9]. The corresponding regulatory elements are expected to drive expression of the transgene preferentially in type IIB fast twitch muscle fibers (affected predominantly in callipyge sheep) throughout pre- and postnatal development [16]. The 7.6 Kb insert was released from the remainder of the vector by digestion with *Not*I and *Sma*I, gel-purified and microinjected into fertilized inbred FVB/N murine eggs, which were re-implanted into pseudo-pregnant foster mothers. Among 41 offspring, eight had genomic integration of the transgene. Six out of the eight founders transmitted the transgene to their offspring. We performed RT-PCR to amplify the *oPEG11* ORF using total RNA extracted from *quadriceps femoris* muscle of two offspring per transmitting founder. Transgene expression was observed for two mouse lines (referred to as 126 and 127; data not shown) and these were analyzed further.

**Fig 1:**
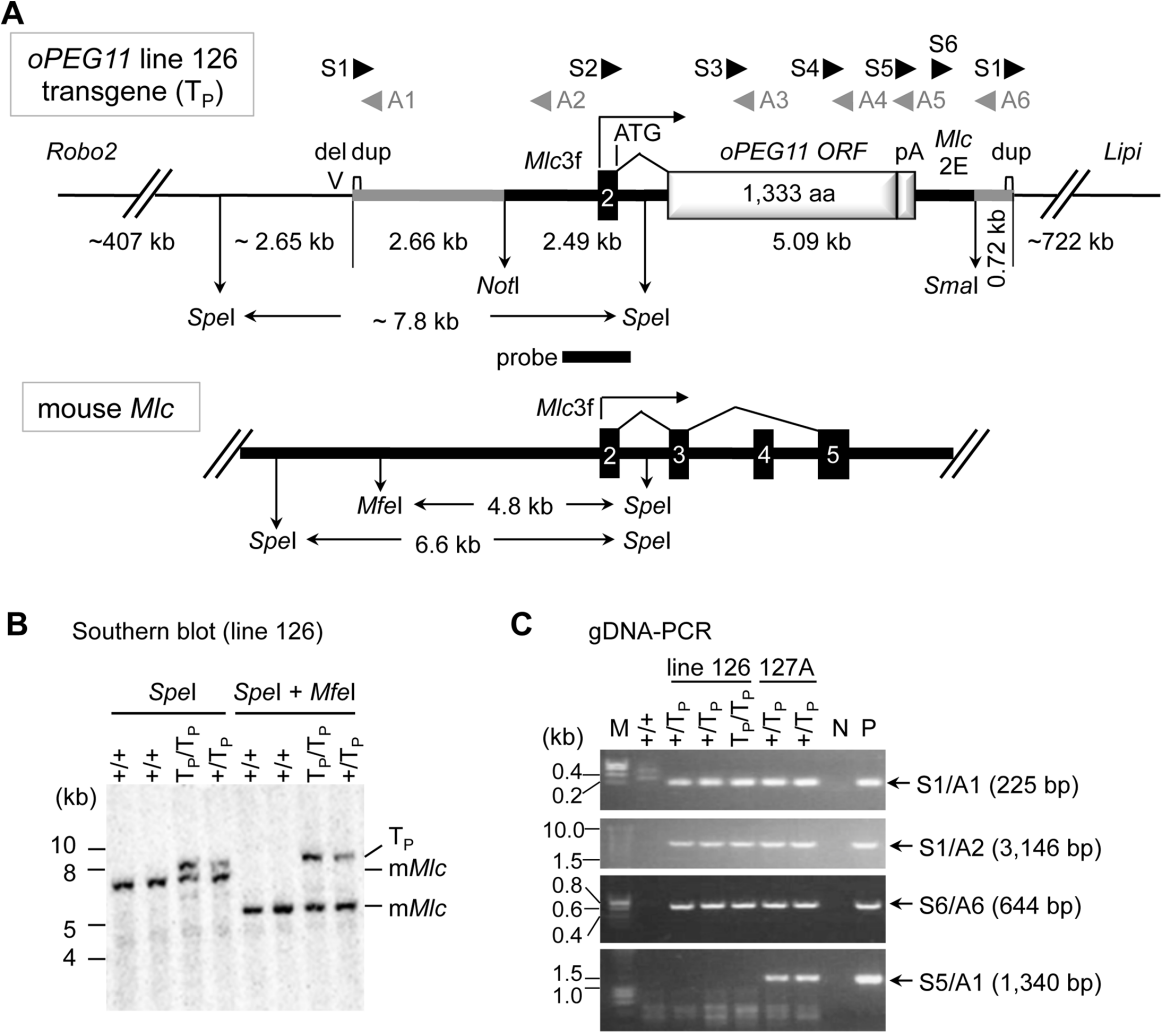
Characterization of the *oPEG11* transgene. **A.** Organization of the *oPEG11* transgene (T_P_) integration site in mouse *oPEG11* line 126 and endogenous mouse *Myosin light chain* (*Mlc*) locus. Thick black lines correspond to upstream, intronic and downstream sequences of the *Mlc* gene including the 3F promotor (Mlc3F) and 2E enhancer (Mlc2E); the numbered black boxes to *Mlc* exons; the thick grey line to vector-specific sequences including a 220 bp duplicated segment marked by a small white box (dup); the light gray boxes to the open reading frame of ovine *PEG11* (*oPEG11* ORF) and bovine *GH* polyadenylation site (pA), respectively; the thin black lines to the transgene integration site flanked by *Robo2* at 407 Kb on the proximal site, and *Lipi* at 722 Kb on the distal site. The positions of the *Not*I and *Sma*I sites in the vector intended to excise the insert DNA for microinjection are shown, as are the positions of the *Spe*I and *Mfe*I sites determining the size of the restriction fragments detected with the indicated probe (probe) in Southern blotting for the transgene integration site and endogenous *Mlc* gene. The position of greater than or equal to 1.6 Kb deletion at the integration site is indicated (del). The position of the primers used for PCR and RT-PCR are shown. **B.** Results of Southern blotting of genomic DNA of homozygous +/+, heterozygous +/T_P_, and homozygous T_P_/T_P_ mice (*oPEG11* line 126), digested with *Spe*I (left) or *Spe*I *+ Mfe*I (right), using the probe with position as shown in A. The bands corresponding to the transgene (T_P_), and endogenous *Mlc* gene (*mMlc*) are marked. **C.** Results of PCRs performed using genomic DNAs and the primers shown in A to clarify the transgene organization in mouse *oPEG11* lines 126 and 127A.

We combined Southern blotting, regular PCR, and “splinkerette” PCR [17] to characterize the transgene insertions in mouse lines 126 and 127. Line 126 was shown to harbor a single-copy transgene that was integrated into an intergenic region on chromosome 16, at ∼407 Kb from *Roundabout homolog 2* (*Robo2*) and ∼722 Kb from *Lipase*, *member I* (*Lipi*) (Fig 1; S1 Fig). Unexpectedly, the transgene comprised both the *Mlc/oPEG11* insert, as well as the remainder of the vector including a 0.22 Kb duplicated segment flanking the transgene on either end (Fig 1). This indicated that the microinjected insert preparation was contaminated with undigested vector. The restriction patterns obtained by Southern blotting suggest that the transgene insertion was accompanied by a more than or equal to 1.6 Kb deletion at the integration site (Fig 1).

**Fig 2:**
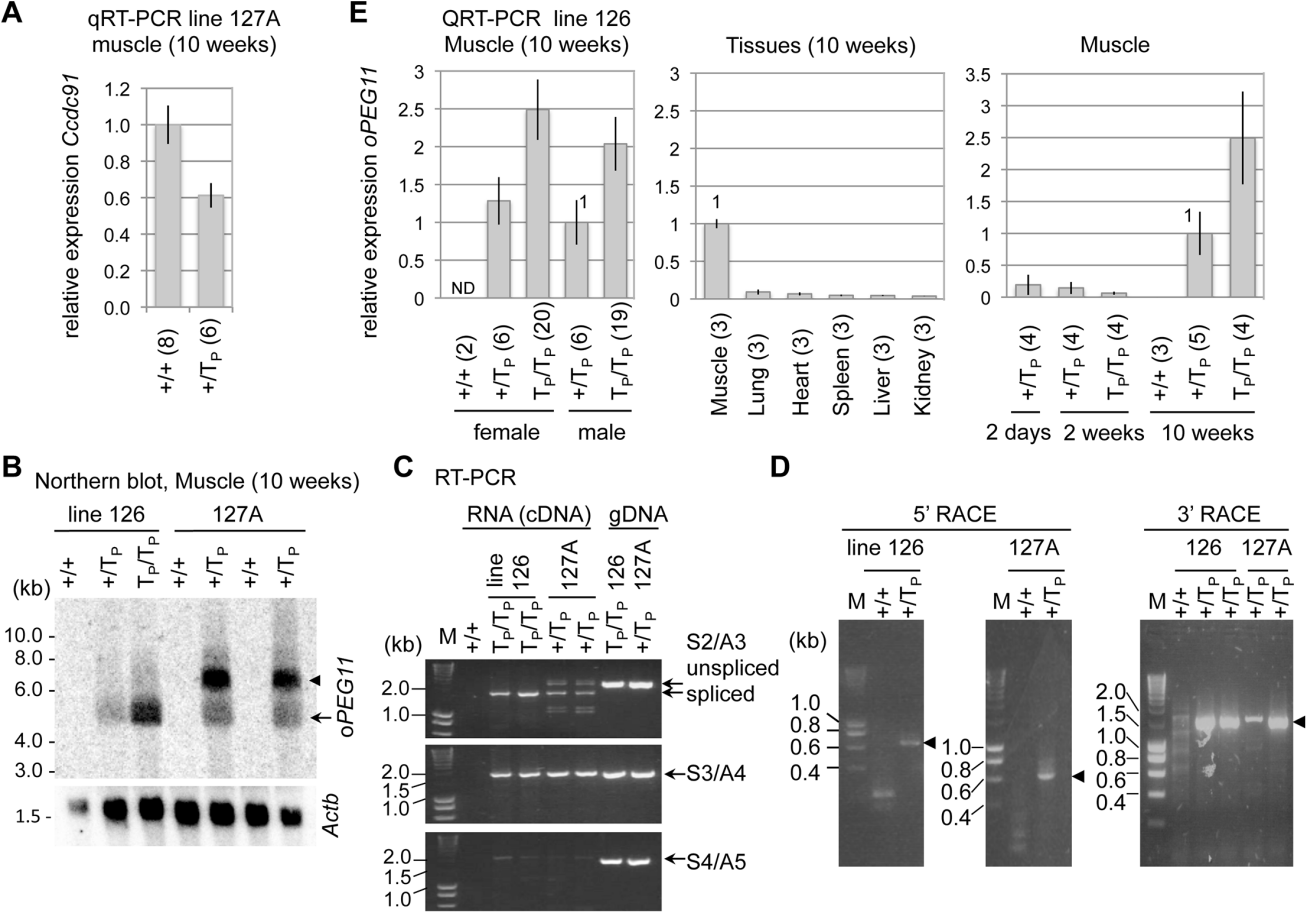
Expression analysis of the *oPEG11* transgenes. **A.** Relative expression levels of *Ccdc91* (mean ± 1.96 x standard error of the mean (s.e.m.)) measured by QRT-PCR using RNA extracted from *quadriceps femoris* muscles of +/+ and +/T_P_ mice from *oPEG11* line 127A. The nearly 1.5-fold reduction in +/T_P_ animals suggests an inactivation of the *Ccdc91* gene and likely explains the absence of T_P_/T_P_ mice in this cross. **B.** Northern blotting of mRNA extracted from *quadriceps femoris* of +/+, +/T_P_, and T_P_/T_P_ mice from *oPEG11* lines 126 and 127A using RNA probes corresponding to the ovine *PEG11* transgene (*oPEG11*) and mouse *β–actin* for control (*Actb*). The bands have the approximate size expected for transcripts derived from the *oPEG11* transgene (4.3 Kb, arrow) in the both mouse lines plus a larger band (~7 Kb, arrow head) in line 127. **C.** RT-PCR (or PCR) performed using total RNA (or DNA) extracted from *quadriceps femoris* (or tail) of +/+, +/T_P_, and T_P_/T_P_ mice from lines 126 and 127A using primer combinations shown in Fig 1A. The extra-bands corresponding to unspliced transcripts observed in line 127A are differentiated from the bands expected for spliced transcripts observed in lines 126 and 127A. **D.** Results of 5’ and 3’ RACE experiments conducted with total RNA extracted from *quadriceps femoris* of +/+, and +/T_P_ mice from lines 126 and 127A. Sequencing the PCR products marked by the arrows confirmed the use of the vector-specific transcription start and polyadenylation sites (data not shown). **E.** Relative expression level of *oPEG11* (mean ± 1.96 x s.e.m.) in line 126 measured by QRT-PCR using total RNA extracted from *quadriceps femoris* from animals of indicated genotypes and ages, and from tissues at 10 weeks of age. Expression levels of +/T_P_ animals were used for references. The numbers of tested samples are shown in parentheses.

Mouse line 127 was shown to harbor two distinct integration sites (127A and 127B), both comprising a similar, complex array thought to encompass multiple tandem copies of digested insert and undigested full-length vector, confirming the contamination of the insert preparation with undigested vector (Fig 1C; S2 Fig). Expression of the transgene was only observed in mice with the 127A integration, not in mice with the 127B integration (data not shown). The 127A integration site was shown to correspond to the last intron of *Coiled-coil domain containing 91* (*Ccdc91*) on chromosome 6 (S1 Fig). *Ccdc91* was shown to be highly expressed in skeletal muscle, and its expression to be reduced strongly by the transgene insertion (p = 5.6x10^-5^; Fig 2A).

We used RT-PCR, rapid amplification of cDNA ends (RACE), and Northern blotting to analyze further the transgene expression in mouse lines 126 and 127A. Northern blotting with total RNA extracted from *quadriceps femoris* of 10-week old animals revealed transgene-specific bands of expected size (∼4.3 Kb) and intensity (∼2:1 homozygote:heterozygote ratio) in line 126. In addition to the same band, a stronger and larger (∼7 Kb) band was observed in line 127A (Fig 2B). RT-PCR analysis with overlapping amplicons encompassing the complete *oPEG11* ORF revealed amplification products of expected size and sequence for line 126 (Fig 2C). Similar results were obtained for line 127A with, however, extra amplification products for the 5’ amplicon (S2/A3 in Fig 2C). The largest of these was shown by sequencing to result from retention of the construct’s intron (733 bp between *Mlc* exon 2 and *oPEG11* ORF) introducing premature termination codons. 5’ and 3’ RACE uncovered the expected, vector-dependent transcription start and polyadenylation sites in both lines (Fig 2D).

Because of the complex structure of the transgene insertion, the occurrence of multiple abnormal *PEG11* transcripts, and the deleterious effect on *Ccdc91* expression precluding the attainment of homozygous mice (data not shown) in line 127A, we decided to pursue detailed analysis of line 126 only. RT-PCR analysis indicated that the line 126 transgene was expressed preferentially in skeletal muscle, and that expression increased from birth to 10 weeks in this tissue (Fig 2E).

### Ectopic expression of ovine *PEG11* causes a muscular hypertrophy in transgenic mice

**Fig 3:**
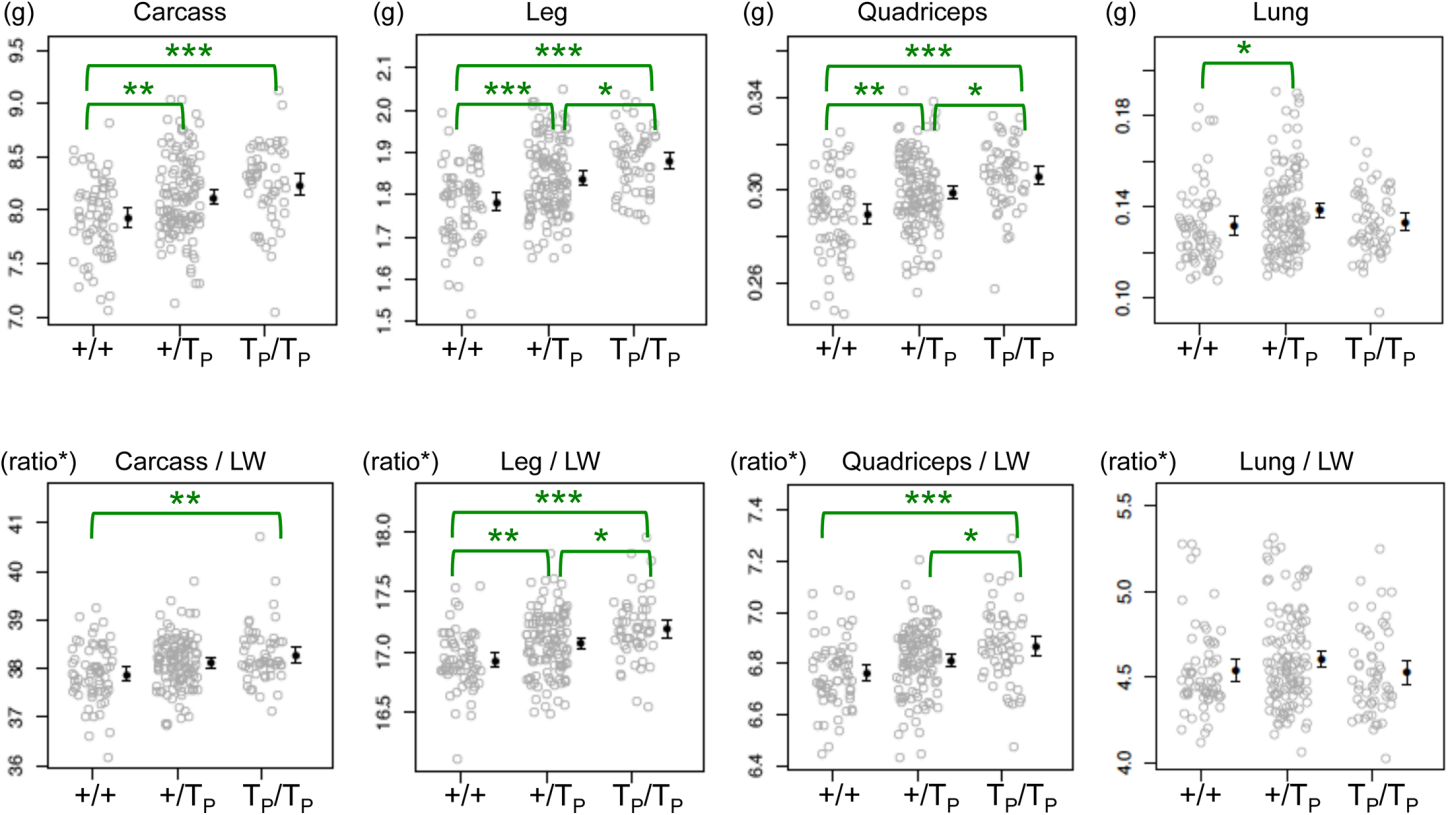
Phenotypic effects of one (+/T_P_) and two (T_P_/T_P_) copies of the *oPEG11* transgene (line 126) in the *oPEG11* F1 mouse cross (+/T_P_ x +/T_P_) at 10 weeks of age. The eight graphs show the distribution of individual phenotypic values corrected for sex and litter effects (carcass weight, the weight of the hind legs, *quadriceps* and lung and their ratios to live weight (LW)) of mice sorted by transgene genotype. Ratios were subject to angular transformation. Genotype means are shown by black dots with error bars (± 1.96 s.e.m.). Statistically significant differences between the possible combinations of genotype computed with the mixed model and Tukey honest significant difference test are marked by braces; ***: p < 0.001, **: p < 0.01, *: p < 0.05 (S2 Table); n = 68 (+/+), 122 (+/T_P_) and 55 (T_P_/T_P_).

Sixteen F1 pairs carrying the *oPEG11* line 126 transgene in a pure FVB background were intercrossed to generate 245 F2 offspring from 28 litters. The average number of offspring per litter was 9, ranging from 4 to 12, hence within the normal range for FVB mice. Sex ratios and genotype frequencies (+/+, +/T(ransgene)_P_ and T_P_/T_P_) did not deviate significantly from expectations (S1 Table). All animals were weighted weekly after weaning, euthanized at ~10 weeks of age (range: 70–74 days) and dissected. For each animal, we recorded live weight, carcass weight, the weight of the hind legs, *quadriceps femoris* muscles, *triceps brachialis* muscles, kidney, spleen, liver and heart. The weight of the hind leg is primarily determined by the mass of the muscle (85.6 ± 1.9%, n = 3). Phenotypic values were analyzed with a mixed model including sex and genotype as fixed effects, and litter as random effect (Model-1, S2 Table). Sex-by-genotype interactions were considered but never significant and therefore not included in the model. Transgene genotype significantly affected the weight of the carcass (p = 1.5x10^-4^), hind legs (p = 2.0x10^-7^), *quadriceps* (p = 2.8x10^-6^), and *triceps* (p = 2.5x10^-3^) (Fig 3; S2 Table). More specifically, one and two copies of the transgene increased carcass weight by 2.3% (p = 6.4x10^-3^) and 3.7% (p = 7.4x10^-5^), and the weight of the hind legs by 3.0% (p = 2.6x10^-4^) and 5.2% (p = 3.4x10^-8^), respectively. For individual muscles the corresponding increases were 3.0% (p = 1.4x10^-3^) and 5.3% (p = 1.4x10^-6^) for *quadriceps*, and 1.7% (not significant) and 3.7% (p = 1.3x10^-3^) for *triceps*.

Transgene genotype marginally increased live weight at 10 weeks of age (p = 0.03), as well as the weight of organs (kidney and lung, p > 0.04), although these effects were not significant when accounting for the realization of multiple tests (p ≥ 0.27 after Šidák correction, S2 Table). To verify further whether the effect on muscularity might just reflect a systemic growth effect rather than being genuinely muscle-specific, we tested the effect of transgene genotype on the weight of individual body parts relative to live weight either by dividing the phenotypic values by live weight (Model-1) or by adding live weight as a covariate in the mixed model (Model-2, S2 Table). The effect on hind legs and *quadriceps* remained highly significant (Šidák corrected p ≤3.7x10^-3^). The effect on organs, however, became non-significant (Šidák corrected p ≥ 0.13) and their relative weight didn’t increase consistently with transgene copy number (Fig 3; S2 Table).

**Fig 4:**
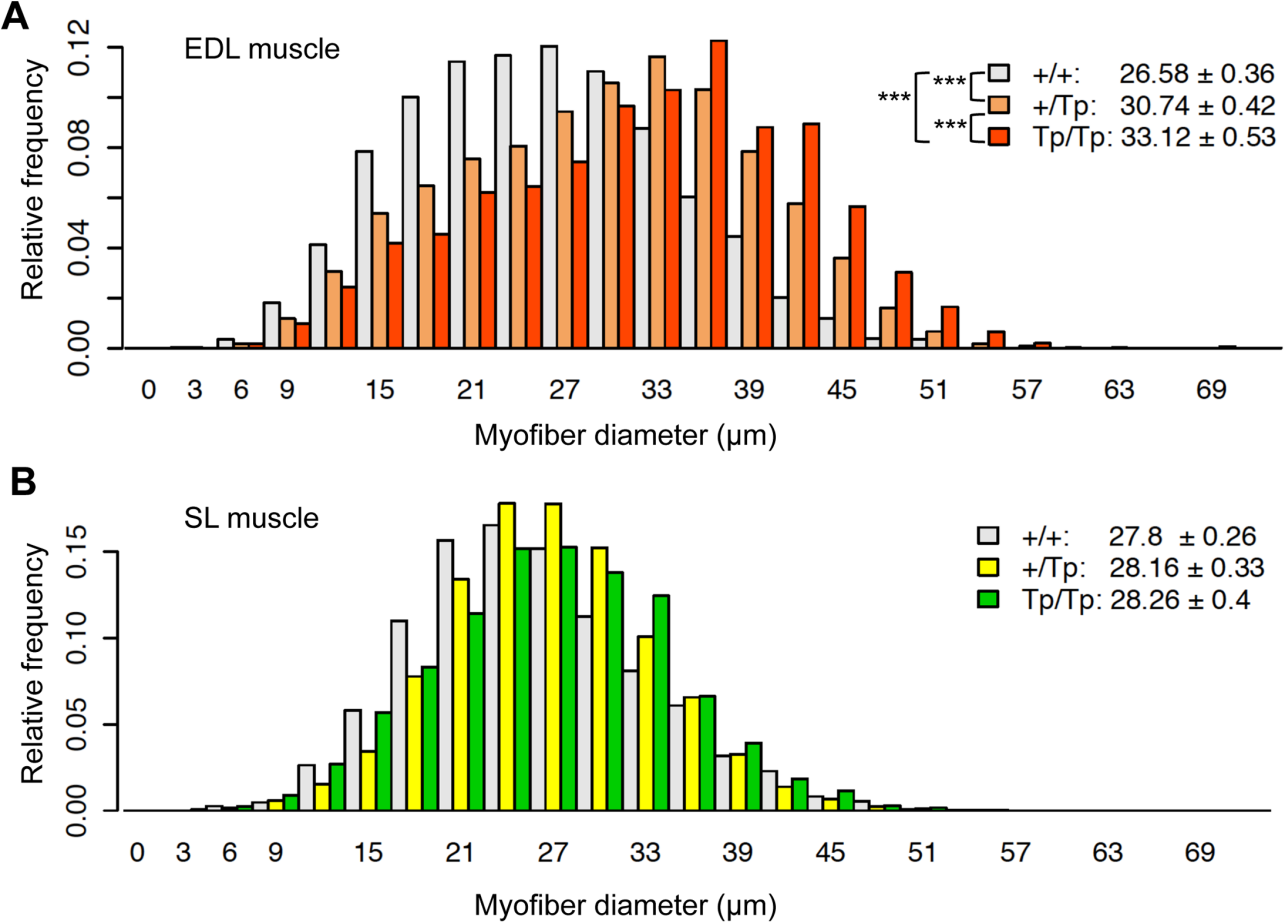
Relative frequency distribution of myofiber cross-sectional size of *extensor digitorum longus* (A) and *soleus* (B) muscle sorted by transgene genotype. The minimal Feret’s diameters of on average 1,062 fibers were measured for each animal-muscle type combination so as to cover the entire cross-sectional area of the muscle. Y-axis shows means of relative frequencies among animals with the same genotype. X-axis shows myofiber cross-sectional size ranging from 0 to 75 μm with a bin width 3. Color used for each genotype class, genotype means of myofober diameter (± 1.96 x s.e.m.) and statistical significance of the contrasts between genotypes computed with the Tukey honest significant difference test are shown in the upper right corner of each Fig ***: p < 0.001; n = 3 (+/+), 5 (+/T_P_) and 2 (T_P_/T_P_).

We next examined the effect of the *oPEG11* transgene on myofiber size distribution. In callipyge sheep, myofiber diameter was increased by 24%, 20% and 7% in *longissimus*, *gluteus medius* and *supraspinatus* muscles, respectively [18]. The ovine DLK1 transgenic mice exhibited increase of the *quadriceps* myofiber diameter by 7.4%, 8.7% and 10.5% in +/T and T/T (line A), and +/T (line D) genotypes, respectively [9]. We here selected to use *extensor digitorum longus* (EDL) and *soleus* (SL) muscles that are known to be rich and poor in type IIB myofibers, respectively (55.7–86.6% for EDL, 0.0–3.1% for SL [19, 20]) where the *Mlc* 3F/2E regulatory elements are expected to be most active [16]. We dissected the two muscles from ten male mice in two litters at 7 and 10 months of age (n = 3, 5, 2 for +/+, +/T_P_ and T_P_/T_P_, respectively) and performed hematoxylin and eosin staining of transverse sections. We measured the minimal Feret’s diameter of myofibers [21] (on average 1,062 myofibers per animal-muscle combination) so as to cover the entire cross-sectional muscle area (Fig 4; S3 Fig). EDL myofiber size was increased by 15.7% and 24.6% in +/T_P_ and T_P_/T_P_ mice, respectively, compared to +/+ (p < 2.0x10^-16^). The contrast between +/T_P_ and T_P_/T_P_ genotypes was also significant (+7.7%, p < 2.0x10^-16^). On the other hand, none of the corresponding contrasts in SL muscle was significant (p > 5.9x10^-2^), consistent with the lower contribution of type IIB fibers in SL muscle. Skewness of the myofiber diameter distribution in EDL muscle was changed from right- to left-tailed by increasing the transgene copy number (+/+ = 0.22, +/T_P_ = -0.05, T_P_/T_P_ = -0.17). This implies that part of the myofibers in EDL muscle (supposedly glycolytic fast twitch fibers) is enlarged by the *oPEG11* transgene expression. We did not observe any apparent histological abnormality in these samples (f.i. necrosis, fibrosis, fat deposition, increase of myofibers with centrally located nuclei) (S3 Fig).

**Fig 5:**
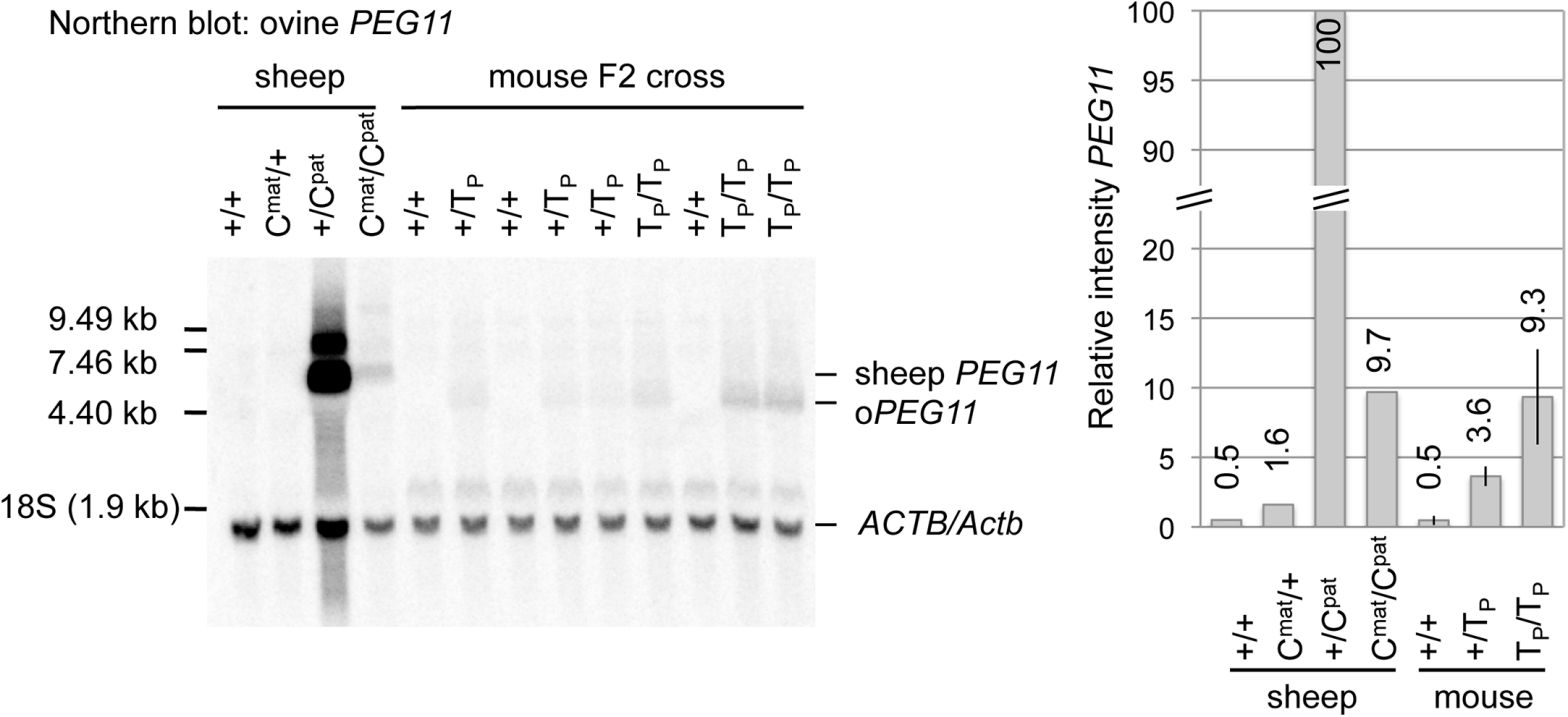
Comparison of *PEG11* expression levels in sheep (at 8 weeks of age) and transgenic mice (at 10 weeks of age). Northern blotting performed with 20 μg of total RNA extracted from *longissimus dorsi* muscles of sheep of the four possible CLPG genotypes (+/+, *CLPG* ^*Mat*^ */+* ^*Pat*^, *+* ^*Mat*^ */CLPG* ^*Pat*^ and *CLPG/CLPG)*, or *quadriceps femoris* muscles of +/+, +/T_P_, and T_P_/T_P_ mice from *oPEG11* line 126 and probed with a strand-specific RNA probe corresponding first to ovine *PEG11*, and secondly to mouse *β–actin*. The bands corresponding presumably to the endogenous ovine *PEG11* (sheep *PEG11*), transgene-derived ovine *PEG11* (*oPEG11*), or *β–actin* (*ACTB/Actb*) are marked. Relative quantities were estimated by densitometric analysis of the autoradiograms (a bar graph on the right) suggesting that expression levels of ovine *PEG11* are ∼10-fold lower in T_P_/T_P_ mice from *oPEG11* line 126 than in callipyge *+* ^*Mat*^ */CLPG* ^*Pat*^ sheep.

Together, our data indicate that postnatal ectopic expression of ovine *PEG11* in skeletal muscle of transgenic mice enhances muscle growth, and this strongly supports a contributing role for *PEG11* in the muscular hypertrophy of callipyge sheep. The magnitude of the effects observed on muscle weight (less than or equal to 5.3% increase (T_P_/T_P_ *quadriceps*)) are modest when compared to those reported in callipyge sheep (f.i. 18.8–21.0% increase in *quadriceps* [22, 23]). To gain some insights in the possible causes of this discrepancy, we evaluated simultaneously the expression level of ovine *PEG11* in *+* ^*Mat*^ */CLPG* ^*Pat*^ sheep as well as +/T_P_ and T_P_/T_P_ transgenic mice by Northern blotting (Fig 5). While the ovine *PEG11* transcripts were clearly detectable in skeletal muscle of transgenic mice, their abundance (estimated by densitometry) was ∼10-fold lower than in callipyge sheep (mouse T_P_/T_P_ versus sheep *+* ^*mat*^ */C* ^*pat*^, Fig 5), and this, in addition to the absence of ectopic expression of *DLK1*, may explain the differences in terms of effect size between mice and sheep.

Following Vuocolo *et al*. [24], we attempted to demonstrate a change in myofiber type composition in +/T_P_ and T_P_/T_P_ transgenic mice by performing QRT-PCR using *quadriceps* muscle RNA for *Myosin heavy chain 1* (*Myh1*), *Myh2*, *Myh4* and *Myh7*, which are specific for type IIx, IIa, IIb and I myofibers, respectively. We observed no significant effect of transgene genotype on the relative amounts of any one of these myosin heavy chain isoforms (S4 Fig).

## Discussion

The question of whether ectopic expression of *DLK1* and/or *PEG11* is responsible for the callipyge muscular hypertrophy has been a matter of debate for some time. The notion that imprinted genes from the *DLK1-GTL2* domain might affect muscle development is supported by both the observed increase in the number of myofibers with centrally located nuclei and also the enlargement of myofiber cross-sectional area in fetuses with paternal uniparental disomy of mouse chromosome 12 (f.i. [25]), and fetuses inheriting a deletion of the *Dlk1-Gtl2* intergenic germline-derived differentially methylated region (IG-DMR) on their maternal chromosome [26, 27]. However, these models perturb the dosage of at least three genes: *Dlk1*, *Peg11* and *Dio3*. As *DIO3* is not affected by the *CLPG* mutation in callipyge sheep, *DLK1* and *PEG11* remained the two most plausible candidates.

A role for *DLK1* appeared well established when it was shown in transgenic mice that ectopic expression of ovine *DLK1* (transmembrane C2 isoform) in pre- and postnatal skeletal muscle caused a muscular hypertrophy [9]. However, the validity of this conclusion was challenged by the observation that BAC-transgenic mice with double or triple *Dlk1* dosage did not exhibit the expected skeletal muscle anomalies [28]. This discrepancy may be explained in part by the fact that the natural spatio-temporal gene expression profile is maintained in the da Rocha *et al*. [28] model, but not in the Davis *et al*. [9] model, in which *DLK1* is re-expressed in a tissue (muscle) and at a developmental stage (postnatal) where it is normally silenced completely.

Direct evidence for a role of *PEG11* in modulating muscle phenotype has not been reported previously. Sekita *et al*. [14] generated transgenic mice overexpressing *Rtl1* (*Peg11*) alone (respecting its normal spatio-temporal expression pattern), but did not report any effect on skeletal muscle. It is noteworthy that the majority of these animals died before or just after birth with severe placental anomalies, and that–in surviving animals–*PEG11* expression would likely have rapidly been downregulated in skeletal muscle. In this study, although presenting results for one line only (*oPEG11* mouse line 126), we strongly believe that the observed increase in muscle mass is due to ectopic expression of the *oPEG11* transgene in skeletal muscle rather than to an unintended side-effect of the transgene insertion. First of all, the phenotype is the most prominent where (skeletal muscle) and when (after 7 weeks of age) the *Mlc* 3F/2E promoter and enhancer is expected to be most active and is independent of live weight [16, 19]. Secondly, we observe a strong effect on the diameter of myofibers in the fast twitch muscle. Thirdly, the integration site is in the middle of a large intergenic region at more than 400 Kb from the closest annotated gene.

Our data indicate that ectopic expression of PEG11 controlled by the *Mlc*3f/2E promoter and enhancer elements in mice affects muscle growth and development, but the precise molecular mechanisms remain elusive. *PEG11/RTL1* is a *sushi*-*ichi*-related retrotransposon homolog and predicted to have a gag (capsid-like), an aspartyl protease, and two transmembrane domains [29, 30]. PEG11 appears to be preferentially localized proximal to nuclei of the endothelial cells of fetal capillaries in the labyrinth zone of placenta [14], and associated with the nuclear fraction in skeletal muscle of callipyge sheep [11]. It has been speculated that the hepatocarcinogenic effect of PEG11 overexpression might result from cleavage of an extracellular matrix component mediated by a speculative aspartyl protease activity of PEG11 [15]. Possible connections, if any, between these observations and an effect on muscle mass are unclear.

In conclusion, this work provides the first direct evidence that excess PEG11 as well as DLK1 in post-natal skeletal muscle contributes to the muscular hypertrophy of callipyge sheep. Additional transgenic mouse lines expressing PEG11 and/or DLK1 at levels equivalent to callipyge sheep would facilitate further analyses to elucidate the mechanisms underlying the muscular hypertrophy in adult skeletal muscle. That might provide new insight useful for animal breeding as well as finding new therapeutic targets to treat muscle loss related to disease and age.

## Materials and Methods

### Oligonucleotide information

All oligonucleotide sequences are listed in S4 Table.

### Transgene construction

The complete ovine *PEG11* ORF was amplified by PCR as seven partially overlapping fragments from BAC 229G11 [6, 31, 32]. These were digested at endogenous restriction enzyme sites (*Bspe*I, *Bgl*I, *Nsi*I, *Aat*II, *BspH*I, *Mlu*I) to generate paired cohesive ends which allowed for the reconstitution by ligation of a 4,178 bp fragment spanning from codon two to 175 bp downstream of the TGA stop codon of the ovine *PEG11* gene (o*PEG11*). The corresponding fragment was digested with *Bgl*II and ligated in frame with the vector’s ATG initiation codon of the pMlc3F-nlacZ-2E vector [9]. A 7.5 Kb fragment encompassing *Mlc* 3F promotor, o*PEG11* ORF, bovine *GH* polyadenylation signal and *Mlc* 2E enhancer was released from the remainder of the vector by digestion with *Not*I and *Sma*I, subjected to agarose gel electrophoresis, and purified with the Geneclean Spin kit (MP Biomedicals, Santa Ana, CA).

### Generation of transgenic mice

Transgenic mice were generated as described previously by Davis *et al*. [9]. Briefly, the purified DNA fragment was microinjected into the pronuclei of fertilized inbred FVB/N (Charles River, Wilmington, MA) mouse eggs at the concentration of 1 ng/μl via standard techniques. Injected eggs were then surgically implanted into pseudopregnant B6CBAF1 (Charles River) foster mothers. The transgene-positive founders were mated with wild type FVB mice to confirm transmission of the transgene upon PCR analysis of tail DNAs from their offspring. To evaluate transgene expression, we performed RT-PCR targeting the o*PEG11* ORF using total RNA extracted from *quadriceps femoris* skeletal muscles of two offspring per transmitting founder.

### Ethical statement

All animal procedures were carried out in strict accordance with the recommendations in the Guide for the Care and Use of Laboratory Animals of University of Liège and approved by the Animal Ethics Committees of University of Liège (Permit Number: LA1610002).

### Characterizing the transgene insertion sites

#### Southern blotting

Genomic DNA was extracted from mouse spleen using standard procedures; proteinase K digestion followed by phenol/chloroform purification and ethanol precipitation. Ten micrograms of genomic DNA were digested with *Spe*I and/or *Mfe*I overnight at 37°C, and separated on a 0.8% agarose gel. The gel was equilibrated in the transfer buffer (0.4N NaOH and 1M NaCl), and DNA was then capillary-transferred to the Hybond N+ membrane (GE Healthcare, Buckinghamshire, UK). DNA probes for Southern hybridization were obtained by PCR amplifications of the vector used for generating transgenic mouse using primers; PEG11-probe1-S1 and–A1 (mouse line 126), or PEG11-probe2-S1 and–S2 (line 127A). The PCR products were then labeled with [α-^32^P] dCTP with the Prime-It II random primer labeling kit (Agilent, Santa Clara, CA). The membrane was hybridized with approximately 10^6^ cpm/ml of the probe in the ULTRAhyb ultrasensitive hybridization buffer (Ambion, Austin, TX) at 55°C overnight. After washing twice with 2 × SSC and 0.1% SDS buffer at 55°C for 5 min and three times at 60°C for 15 min with 0.1 × SSC and 0.1% SDS, the membrane was exposed overnight to a phosphor screen which was subsequently scanned with the Typhoon 9400 instrument (GE Healthcare).

#### PCR

PCR amplifications using genomic DNAs or the vector DNA were conducted with the Phusion Hot Start II High-Fidelity DNA Polymerase (Finnzymes, Espoo, Finland) according to the manufacturer’s instructions under the following conditions: (1) 98°C 2 min, (2) 5 cycles of 98°C for 10 s, 62°C for 20 s and 72°C for 50 s, (3) 5 cycles of 98°C for 10 s, 60°C for 10 s and 72°C for 50 s, (4) 25 cycles of 98°C for 10 s, 58°C for 10 s and 72°C for 40 s, followed by (5) a 5 min final extension at 72°C. The PCR products were analyzed by electrophoresis on a 1.5% agarose gel.

#### Splinkerette PCR

Splinkerette PCR was performed according to Potter & Luo [17]. Briefly, 1 μg of genomic DNA was digested with *Fat*I (for line 126) or *Apo*I (for line 127A), and ligated to a corresponding linker consisting of double stranded splinkerette oligonucleotides with a stable hairpin loop at one side and a compatible cohesive end at another side (SPLK-A and SPLK-B oligonucleotides were used for *Fat*I digestion, and SPLK-A and SPLK-E for *Apo*I digestion, respectively). Two rounds of nested PCRs were conducted using primers; SPLA1 with either S7 (line 126) or S9 (line 127A) for the first round of PCR, and SPLA2 with either S8 (line 126) or S10 (line 127A) for the second round of PCR. The PCR products were gel-purified and sequenced using a standard Sanger sequencing procedure to determine integration sites of the o*PEG11* transgene in the mouse genome.

### Genotyping assays for mouse lines 126 and 127A

According to the information of the 126 and 127A transgene integration sites, we designed allele-specific PCR assays for both lines (S1 Fig). PCRs targeting wild-type and transgene alleles were performed separately using the Go-Taq DNA polymerase (Promega, Madison, WI) under the following condition: (1) 95°C 3 min, (2) 35 cycles of 95°C for 30 s, 60°C for 30 s and 72°C for 30 s, followed by (3) a final extension at 72°C for 5 min. Amplification products were pooled and detected by electrophoresis on a 2.5% agarose gel.

### Characterizing the expression of *oPEG11* transgenes in mouse lines 126 and 127A at the RNA level

#### RNA extraction

Total RNA was extracted using the Trizol (Invitrogen, Carlsbad, CA) and treated with the Turbo DNase (Ambion) prior to RT-PCR or RACE according to the manufacturer’s instructions.

#### Northern blotting

Northern blotting was performed essentially by following Green and Sambrook [33]. Briefly, 20 μg of total RNA was denatured at 65°C for 15 min in three volumes of the NorthernMax formaldehyde load dye (Ambion) and subjected to electrophoresis on a 1% agarose gel containing 2.4 M formaldehyde in 1 x MOPS buffer (Lonza, Basel, Switzerland) at 5 V/cm for ~5 h. The gel was soaked in 0.01 N NaOH/3 M NaCl for 20 min, and RNA was transferred to the Hybond N+ membrane using upward capillary flow of 10 × SSC buffer (150 mM sodium citrate, 1.5 M NaCl, pH 7.0) for 1 hr. RNA was cross-linked on the membrane using the Stratalinker UV crosslinker (Stratagene, La Jolla, CA). For preparing probes, part of *oPEG11* ORF (199-bp) was PCR-amplified using sheep muscle cDNA and primers (antisense primers were appended with a T7 RNA polymerase promoter), ovPEG_P_sen1 and _ans1_T7, and purified with the DNA Clean & Concentrator-5 (Zymo research, Irvine, CA). RNA probe was synthesized using the MAXIscript kit (Ambion) from one microgram of the PCR product with T7 RNA polymerase with [α-^32^P] UTP according to the manufacturer’s instructions. After DNase I treatment and column purification using the ProbeQuant G-50 micro column (GE Healthcare), approximately 1 × 10^6^ cpm/ml of the RNA probe was used for hybridization at 68°C overnight. The membrane was washed twice at 70°C for 10 min in 2 x SSC and 0.1% SDS, then 3~5 times at 70°C for 30 min in 0.1 x SSC and 0.1% SDS, exposed to a phosphor screen for a few days and scanned with the Typhoon 9400 instrument. RNA probe targeting mouse *β–actin* (for control) was prepared from a plasmid DNA supplied in the MAXIscript kit.

#### RT-PCR

cDNA synthesis was performed using 1 μg of total RNA with 40 pmol of oligo (dT)_18_, 40 pmol of random hexamer and 1 pmol each of two *oPEG11*-specific primers (A4 and PEG-cDNA2) using the RevertAid H Minus First Stand cDNA Synthesis kit (Fermentas, Burlington, Canada) according to the manufacturer’s instructions. PCR amplification was performed using the Phusion Hot Start II High-Fidelity DNA Polymerase under the following conditions: (1) 98°C 1 min, (2) 5 cycles of 98°C for 10 s, 65°C for 20 s and 72°C for 90 s, (3) 5 cycles of 98°C for 10 s, 60°C for 10 s and 72°C for 80 s, (4) 25 cycles of 98°C for 10 s, 58°C for 10 s and 72°C for 70 s, followed by (5) 5 min final extension at 72°C. The PCR products were purified after electrophoresis on 1.5% agarose gels and validated by sequencing.

#### qRT-PCR

cDNA was synthesized from 1 μg of total RNA using 50 pmol each of oligo (dT)_18_ and random hexamers. QRT-PCR assay was performed using the Absolute Blue QPCR SYBR Green ROX Mix (ABgene, Surrey, UK) according to the manufacturer’s instructions on the 7900HT Real-Time PCR System (Applied Biosystems, Foster City, CA) under the following conditions: (1) initial denaturing at 95°C for 15 min, followed by (2) 40 cycles of 95°C 15 s, 60°C 30 s, and 72°C 30 s, and (3) a melting curve analysis with 95°C 15 s, 60°C 15 s, and 95°C 15 s. Expression levels of target genes were normalized relatively to the expression levels of two control genes (*Hypoxanthine phosphoribosyltransferase 1* and *Actin gamma 1*) that were selected out of five tested housekeeping genes (*Splicing factor 3a subunit 1*, *Actin beta*, *Topoisomerase DNA II beta*) using 2- and 10-week old mouse *quadriceps* muscles according to Vandesompele *et al*. [34].

#### RACE

cDNAs for 5’ and 3’ RACE were synthesized with the GeneRacer Kit (invitrogen) from five and one micrograms of total RNA, respectively, according to the manufacturer’s instructions. To identify a 5’ end of transcript (5’ RACE), cDNA was amplified using GeneRACE 5’ and *oPEG11*-specific 5GSP-O primers for the first round of PCR, and GeneRACE 5’ Nested and *oPEG11*-specific 5GSP-I primers for the second round of PCR. To identify a polyadenylation site (3’ RACE), GeneRACE 3’ and *oPEG11*-specific 3GSP primers were used. The PCR amplification was performed by using the Phusion Hot Start II High-Fidelity DNA Polymerase under the following conditions: (1) 98°C 30 s, (2) 4 cycles of 98°C 10 s, 70°C 30 s, (3) 4 cycles of 98°C 10 s, 68°C 30 s, (4) 25 cycles of 98°C 10 s, 65°C 10 s, 72°C 30 s and (5) a final extension at 72°C for 5 min. The RACE products were gel-purified after electrophoresis and sequenced by standard procedures.

### Phenotyping

Mice were weaned at 21 days of age and kept with 4~5 mice per cage. Mice were weighted at weaning and then weekly until 10 weeks of age. Animals were euthanized at 10 weeks of age (range: 70–74 days) and dissected. We measured carcass (corresponding to skinned body with head, ankles, tail, internal organs, and associated fat removed), hind legs (corresponding to skinned limbs between hip and ankle), *quadriceps femoris* muscles, *triceps brachialis* muscles, kidney, spleen, liver and heart weights. Experimentalists were unaware of the animals’ genotypes upon dissection.

### Statistical analysis of mouse phenotype

Data points for which the residuals (i.e. phenotype corrected for fixed and random effects) were greater or equal to 3 or less than or equal to -3 times the residual standard deviation were considered as outliers and omitted from further analyses. Ratios (phenotypes divided by live weight) were subject to angular transformation (i.e. $(\arcsin\sqrt{ratio})\times 180÷\pi$) prior to analysis. Phenotypic values were analyzed with a mixed model including sex and genotype (and live weight) as fixed effects and litter as random effect. The effect of genotype accounted for multiple comparisons was calculated with the Šidák correction. Effects in possible contrasts of genotypes were computed with the Tukey honest signifcant difference test. Statistical analyses were conducted with R [35] (packages: lme4 [36], lmerTest [37], multcomp [38]) and SAS (SAS, Cary, NC).

### Morphometric analysis

*Extensor digitorum longus* (EDL) and *soleus* (SL) muscles were dissected from male mice at 7 and 10 months of age. Both muscles from an individual were pinned up on a cork plate, fixed in 4% formaldehyde at room temperature for approximately four hours, cut at the midpoint perpendicularly to the long axis of the muscles, and embedded in a paraffin block so that the cutting planes were oriented for sectioning. Transverse sections (two sections per muscle) were prepared at a thickness of 5 μm and stained with hematoxylin and eosin by a standard procedure. Digital images of the entire muscle cross-sectional area were acquired at 20x magnification using the FSX100 microscope (Olympus, Center Valley, PA). To estimate myofiber cross-sectional size, approximately 500 myofibers per section so as to cover the entire area of the muscle were manually framed and their minimal Feret’s diameters were measured as a geometrical parameter for myofiber cross-sectional size [21] using the ImageJ software [39]. The fiber diameter was analyzed using a linear regression model containing genotype as a fixed effect and its statistical significance of contrasts between genotypes was computed using the Tukey’s honest significant difference method using R (package stat [40]).

## Supporting Information

S1 Fig Identification of transgene integration sites and genotyping of mice. **A&C.** Results of “splinkerete PCR” experiments conducted to identify the genomic sequence flanking the transgenes in mouse lines 126 and 127A, respectively. Sequencing the PCR products yielded the highlighted sequences (red: transgene-specific sequences; blue: mouse sequences at the integration sites) located in the genome by BLAT analysis. The corresponding locations are shown on screen captures of the UCSC genome browser. **B&D.** Results of the genotyping tests generated for lines 126 and 127A, respectively, on the basis of the obtained integration sites as described in Materials and Methods, and allowing unambiguous discrimination of the three possible genotypes.
(TIF)

S2 Fig Characterization of *oPEG11* transgene in mouse line 127. **A.** Organization of the *oPEG11* transgene (T_P_) integration site and endogenous *Myosin light chain* (*Mlc*) gene in mouse lines 127A and 127B. The thick black lines correspond to upstream, intronic and downstream sequence of the *Mlc* gene including the 3F promotor (*Mlc*3F) and 2E enhancer (*Mlc*2E); the numbered black boxes to *Mlc* exons; the thick grey line to vector-specific sequences; the light gray boxes to the open reading frame of ovine *PEG11* (*oPEG11* ORF) and bovine *GH* polyadenylation site (pA) respectively. The positions of the *Not*I and *Sma*I sites in the vector intended to excise the insert are shown, as are the positions of the *Spe*I sites determining the size of the restriction fragments detected with the indicated probe (probe) in Southern blotting for the transgene integration site and endogenous *Mlc* gene. The position of the primers used for PCR and RT-PCR (Figs 1C and 2C) are shown and labeled as in S4 Table. **B.** Results of Southern blotting of genomic DNA of +/+, +/A, +/B, and A/B mice, digested with *Spe*I, using the probe with position as shown in A. Bands with ∼10.7 Kb corresponding to tandem transgene copies of the type shown in blue in A, ∼7.5 Kb bands corresponding to tandem transgene copies of the type shown in red in A, ∼9.0 Kb and ∼4.1 Kb bands presumably corresponding to transgene copies flanking the tandem repeats, and 3.9 Kb bands corresponding to the endogenous *Mlc*3F genes are marked.
(TIF)

S3 Fig Morphological analyses of *extensor digitorum longus* (EDL) and *soleus* (SL) muscles of the *oPEG11* line 126 F2 mice. (A) Box plots show distributions of the myofiber cross-sectional size (minimal Feret’s diameter) sorted by animal and muscle. Frequency distributions sorted by genotype using the same data are shown in Fig 4. The box plots are colored according to its genotype class as is in Fig 4. Age of animal (month) is shown in parenthesis. n = 3 (+/+), 5 (+/T_P_) and 2 (T_P_/T_P_). (B) Hematoxylin and eosin staining of transverse sections of EDL and SL muscles. Scale bar represents 50 μm. No apparent histological abnormality such as necrosis, fibrosis, fat deposition or increase in myofiber with centrally located nuclei was observed.
(TIF)

S4 Fig RNA expression analysis of *Myosin heavy chain* isoforms. Results of QRT-PCR experiments conducted using *quadriceps femoris* muscle RNA and targeting endogenous mouse *Myosin heavy chain* isoforms *Myh1*, *Myh2*, *Myh4* and *Myh7* to identify putative myofiber type alterations as a result of *oPEG11* transgene expression. None of the changes were statistically significant.
(TIF)

S1 Table Sex-specific and pooled genotypic proportions and associated probabilities under the null hypothesis of Mendelian segregation. (XLSX)

S2 Table Transgene effects in the *oPEG11* (line 126) F2 mice at 10 weeks of age. (XLSX)

S3 Table Live weights of the *oPEG11* (line 126) F2 mice between weaning and 10 weeks of age. (XLSX)

S4 Table Oligonucleotide information. (XLSX)
