# Supplementary figures and images for: Ectopic Expression of Retrotransposon-Derived PEG11/RTL1 Contributes to the Callipyge Muscular Hypertrophy
Source: PLoS One. 2015 Oct 16;10(10):e0140594. doi: 10.1371/journal.pone.0140594 (PMC4608697; doi:10.1371/journal.pone.0140594)

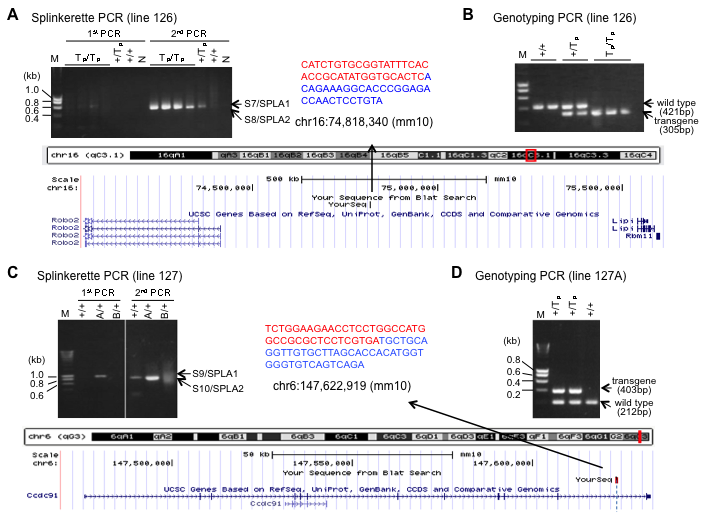

Supplement: S1 Fig — A&C. Results of “splinkerete PCR” experiments conducted to identify the genomic sequence flanking the transgenes in mouse lines 126 and 127A, respectively. Sequencing the PCR products yielded the highlighted sequences (red: transgene-specific sequences; blue: mouse sequences at the integration sites) located in the genome by BLAT analysis. The corresponding locations are shown on screen captures of the UCSC genome browser. B&D. Results of the genotyping tests generated for lines 126 and 127A, respectively, on the basis of the obtained integration sites as described in Materials and Methods, and allowing unambiguous discrimination of the three possible genotypes. (TIF) [file pone.0140594.s001.tif]

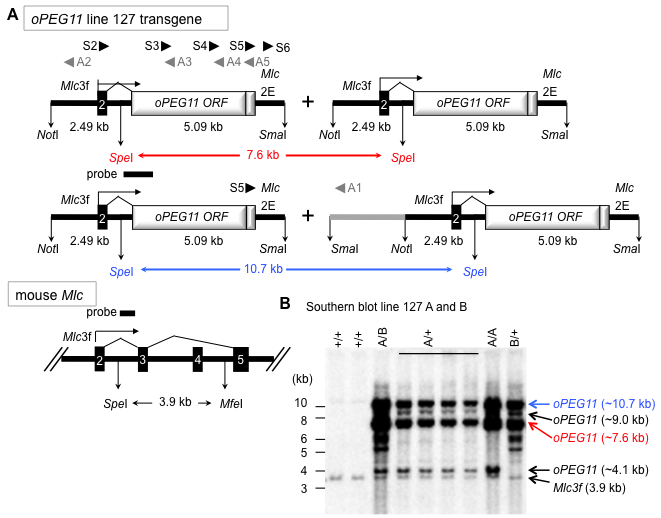

Supplement: S2 Fig — A. Organization of the oPEG11 transgene (TP) integration site and endogenous Myosin light chain (Mlc) gene in mouse lines 127A and 127B. The thick black lines correspond to upstream, intronic and downstream sequence of the Mlc gene including the 3F promotor (Mlc3F) and 2E enhancer (Mlc2E); the numbered black boxes to Mlc exons; the thick grey line to vector-specific sequences; the light gray boxes to the open reading frame of ovine PEG11 (oPEG11 ORF) and bovine GH polyadenylation site (pA) respectively. The positions of the NotI and SmaI sites in the vector intended to excise the insert are shown, as are the positions of the SpeI sites determining the size of the restriction fragments detected with the indicated probe (probe) in Southern blotting for the transgene integration site and endogenous Mlc gene. The position of the primers used for PCR and RT-PCR (Figs 1C and 2C) are shown and labeled as in S4 Table. B. Results of Southern blotting of genomic DNA of +/+, +/A, +/B, and A/B mice, digested with SpeI, using the probe with position as shown in A. Bands with ∼10.7 Kb corresponding to tandem transgene copies of the type shown in blue in A, ∼7.5 Kb bands corresponding to tandem transgene copies of the type shown in red in A, ∼9.0 Kb and ∼4.1 Kb bands presumably corresponding to transgene copies flanking the tandem repeats, and 3.9 Kb bands corresponding to the endogenous Mlc3F genes are marked. (TIF) [file pone.0140594.s002.tif]

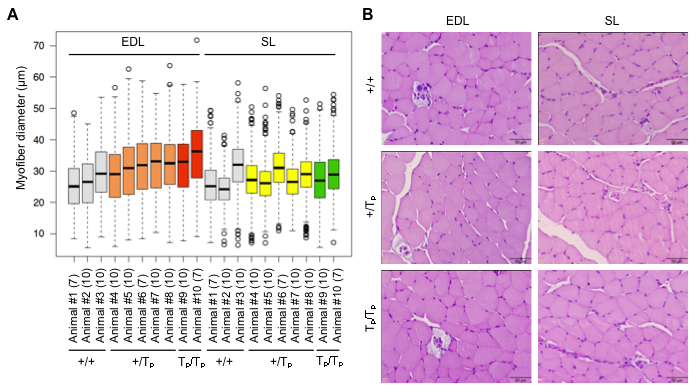

Supplement: S3 Fig — (A) Box plots show distributions of the myofiber cross-sectional size (minimal Feret’s diameter) sorted by animal and muscle. Frequency distributions sorted by genotype using the same data are shown in Fig 4. The box plots are colored according to its genotype class as is in Fig 4. Age of animal (month) is shown in parenthesis. n = 3 (+/+), 5 (+/TP) and 2 (TP/TP). (B) Hematoxylin and eosin staining of transverse sections of EDL and SL muscles. Scale bar represents 50 μm. No apparent histological abnormality such as necrosis, fibrosis, fat deposition or increase in myofiber with centrally located nuclei was observed. (TIF) [file pone.0140594.s003.tif]

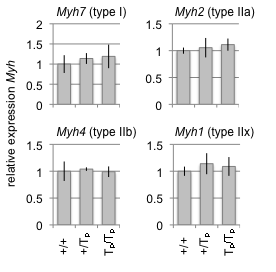

Supplement: S4 Fig — Results of QRT-PCR experiments conducted using quadriceps femoris muscle RNA and targeting endogenous mouse Myosin heavy chain isoforms Myh1, Myh2, Myh4 and Myh7 to identify putative myofiber type alterations as a result of oPEG11 transgene expression. None of the changes were statistically significant. (TIF) [file pone.0140594.s004.tif]
